# Finding the best predictive model for hypertensive depression in older adults based on machine learning and metabolomics research

**DOI:** 10.3389/fpsyt.2024.1370602

**Published:** 2024-06-27

**Authors:** Jiangling Guo, Jingwang Zhao, Peipei Han, Yahui Wu, Kai Zheng, Chuanjun Huang, Yue Wang, Cheng Chen, Qi Guo

**Affiliations:** ^1^ Department of Rehabilitation Medicine, Shanghai University of Medicine and Health Sciences Affiliated Zhoupu Hospital, Shanghai, China; ^2^ Graduate School, Shanghai University of Traditional Chinese Medicine, Shanghai, China; ^3^ Shanghai YangZhi Rehabilitation Hospital (Shanghai Sunshine Rehabilitation Center), School of Medicine, Tongji University, Shanghai, China; ^4^ School of Health Science and Engineering, University of Shanghai for Science and Technology, Shanghai, China; ^5^ School of Health, Fujian Medical University, Fuzhou, Fujian, China

**Keywords:** hypertension, depression, non-targeted metabolomics, GC/LC-MS, machine learning

## Abstract

**Objective:**

Depression is a common comorbidity in hypertensive older adults, yet depression is more difficult to diagnose correctly. Our goal is to find predictive models of depression in hypertensive patients using a combination of various machine learning (ML) methods and metabolomics.

**Methods:**

Methods We recruited 379 elderly people aged ≥65 years from the Chinese community. Plasma samples were collected and assayed by gas chromatography/liquid chromatography-mass spectrometry (GC/LC-MS). Orthogonal partial least squares discriminant analysis (OPLS-DA), volcano diagrams and thermograms were used to distinguish metabolites. The attribute discriminators CfsSubsetEval combined with search method BestFirst in WEKA software was used to find the best predicted metabolite combinations, and then 24 classification methods with 10-fold cross-validation were used for prediction.

**Results:**

34 individuals were considered hypertensive combined with depression according to our criteria, and 34 subjects with hypertension only were matched according to age and sex. 19 metabolites by GC-MS and 65 metabolites by LC-MS contributed significantly to the differentiation between the depressed and non-depressed cohorts, with a VIP value of more than 1 and a P value of less than 0.05. There were multiple metabolic pathway alterations. The metabolite combinations screened with WEKA for optimal diagnostic value included 12 metabolites. The machine learning methods with AUC values greater than 0.9 were bayesNet and random forests, and their other evaluation measures are also better.

**Conclusion:**

Altered metabolites and metabolic pathways are present in older adults with hypertension combined with depression. Methods using metabolomics and machine learning performed quite well in predicting depression in hypertensive older adults, contributing to further clinical research.

## 1 Introduction

Hypertension is a prevalent chronic disease, affecting every third adult worldwide, and constitutes a significant global contributor to disability (1). In China, nearly half of individuals aged 35–75 experience hypertension, with the prevalence increasing with age (2). Despite these alarming statistics, hypertensive patients often grapple with psychological challenges stemming from the prolonged use of antihypertensive medications, diminished quality of life, and physical symptoms (3, 4). Among patients with chronic diseases, depression emerges as the most prevalent mental health disorder (5). In situations where hypertension and depression coexist, the presence of depression can adversely impact adherence to hypertension treatment and further worsen the condition of hypertension (6). Conversely, patients with hypertension combined with depression are more likely to develop further depressive symptoms (7). Moreover, medications used for depression treatment, such as ketamine, can affect the cardiovascular system and increase blood pressure, making it more difficult to treat depression (5, 6, 8). Hence, there is a pressing need for heightened awareness and attention to cases involving the co-occurrence of hypertension and depression. Currently, depression is diagnosed using a scale (9). There are numerous depression diagnostic scales with varying degrees of sensitivity and specificity for diagnosis (10, 11). Therefore, the diagnosis of depression may be misdiagnosed or missed. Consequently, there is an imperative need for an objective and sensitive diagnostic method for depression.

Metabolomics is the study of all small molecule metabolites and chemical processes in organisms and tissues, and is an important tool for discovering changes in metabolic biomarkers in living organisms (12). Metabolomics is widely applied to the study of biomarkers and the study of physiological processes and phenotypic changes associated with disease (13). At present, it has been found through serum and plasma metabolomics that depression can affect changes in metabolites such as blood lipids, amines, neurotransmitters, and amino acids in the blood (14, 15). Metabolic pathways such as glycerophospholipid metabolism, purine metabolism, alanine, aspartic acid, and glutamate metabolism are also affected (16). However, the metabolomics raw data is complex and diverse and poses great challenges in data analysis (13, 17). Therefore, the metabolomics community has always been eager to adopt new mathematical and computational tools to improve data analysis.

Machine learning (ML) can be used to develop models that can handle large-scale data and solve complex problems through learning (12). The application of ML transcends the limitations posed by conventional statistical models, particularly in the realm of metabolomics big data analysis, where the latter often proves inadequate (18). Despite the remarkable potential inherent in the amalgamation of machine learning and metabolomics, the intersection of these domains has been relatively understudied. A previous study has used support vector machine algorithms in machine learning to find diagnostic biomarkers in some indicators commonly measured in hospitals in older adults with hypertensive depression (19). Currently, most of the studies, like the ones mentioned above, ML is widely used to construct models in a number of indicators that are common in hospitals, looking for predictive models of potential biomarkers to diagnose diseases (18). Consequently, the diagnostic capacity of these models may be circumscribed, prompting the need for a more comprehensive exploration of indicators to enhance diagnostic precision.

The goal of this paper is to advance the sensitivity and specificity of the diagnosis of depression in hypertensive patients. In this study, plasma samples were analyzed using gas chromatography (GC) and liquid chromatography (LC) coupled with mass spectrometry (MS), which was able to identify additional plasma metabolites. Subsequently, machine learning techniques were combined with metabolomics to determine the best combination of metabolites and algorithms. This method helps to detect the diagnosis of depression in hypertensive patients out.

## 2 Materials and methods

### 2.1 Study participants

This study recruited 379 participants aged ≥65 who were residents of Shanghai and had participated in China’s nationwide complimentary physical examination initiative (20, 21). A comprehensive geriatric assessment and in-depth face-to-face interviews were conducted. The 30-item Geriatric Depression Scale (GDS) was employed during these interviews to evaluate the presence of depression (22). Simultaneously, we gathered sociodemographic data, disease history, and medication utilization through a meticulous inquiry. Sociodemographic variables comprised age and gender, while chronic conditions included diabetes, hypertension, hyperlipidemia, stroke, and heart disease. Additionally, anthropometric measurements, encompassing height and weight, were taken to calculate the Body Mass Index (BMI). Furthermore, fasting plasma samples were systematically collected from the participants for subsequent metabolomics analyses. The following criteria were used to exclude subjects: incomplete data for our requirements and use of antidepressant medication (23). Nine subjects had incomplete data and two were taking antidepressants. Ultimately, a total of 368 participants met the eligibility criteria and were included in our study. This research received ethical approval from the Ethics Committee at Shanghai University of Medicine and Health Sciences, China, and adhered scrupulously to the principles delineated in the Declaration of Helsinki. All participants provided informed consent before their involvement in the study.

### 2.2 Determination of depression and hypertension

Depression were assessed by the Chinese version of GDS, a standardized self-report questionnaire containing 30 dichotomous questions with good validity and reliability (22). There are 30 items on the scale, either positive or negative. The sum of these 30 items yields a score from 0 to 30, with scores greater than 11 defined as depression (22). Hypertension was defined as a systolic blood pressure of ≥140 mm Hg or diastolic blood pressure of ≥90 mm Hg. The methodology used in this study to consider it as hypertension is the subject’s self-report of having been diagnosed with hypertension by a hospital doctor (24).

### 2.3 Metabolomics analyses

The plasma sample preparation along with LC-MS analysis have been described in detail in our previous study (25). Each plasma specimen was procured from the study participants during a fasting state in the morning and subsequently stored at -80° until analytical scrutiny. For LC-MS analysis, 150 μl of plasma was taken and 10 μl of methanol-solubilized 2-chlorophenylalanine (0.3 mg/ml) was added as an internal standard, along with 450 μl of methanol/acetonitrile (2/1). Vortex for 60 seconds, then sonicate the extract for 10 minutes, let it stand for 30 minutes and then centrifuge for 10 minutes (4°C, 13,000 rpm). 200 μl of supernatant was freeze-concentrated in a centrifugal dryer and then redissolved in 300 μl methanol/water (1/4). The extract was vortexed for 30 seconds, then sonicated for 3 minutes and centrifuged for 10 minutes (4°C, 13,000 rpm). Subsequently, 150 μl of the supernatant was filtered through a 0.22 μm microfilter and transferred to LC vials.

For GC‐MS analysis, 150 μl of plasma was vortexed for 10 seconds with 20 μl of 2-chlorophenylalanine (0.3 mg/ml) dissolved in methanol. Then, 450 μl of ice-cold methanol/acetonitrile (2/1, v/v) was added and vortexed for 30 seconds. The extract was sonicated for 10 min, stored for 30 min (-20°C), and then centrifuged at 4°C for 10 min (13,000 rpm). 200 μl of the supernatant was loaded into a new glass vial and dried in a freeze-concentration centrifuge, after which 80 μL of 15 mg/ml methoxyamine hydrochloride (in pyridine) was added. The resulting mixture was vortexed for 2 minutes and then incubated at 37°C for 90 minutes. 50-μL BSTFA (with 1% TMCS) and 20-μL hexane were added to the bottle, which was then vigorously shaken for 2 minutes and derivatized at 70°C for 60 minutes. The samples were placed at room temperature for 30 min before GC-MS.

LC-MS analysis was conducted utilizing the ACQUITY UPLC I-Class system (Waters Corporation, Milford, USA) coupled with the VION IMS QT high-resolution mass spectrometer (Waters Corporation, Milford, USA). In both positive and negative modes, an ACQUITY UPLC BEH C18 column (1.7μm, 2.1 × 100mm) was employed. For GC-MS analysis, an Agilent 7890B gas chromatography system coupled to an Agilent 5977A MSD system (Agilent Technologies Inc, CA, USA) was utilized. The separation of derivatives was achieved using a DB-5MSf fused-silica capillary column (30m × 0.25mm × 0.25μm, Agilent J& W Scientific, Folsom, CA, USA). Regardless of whether it is an LC-MS or GC-MS analysis, QC samples are added regularly and analyzed every ten samples.

The LC-MS and GC-MS data were initially in an unprocessed form. The processing of LC-MS raw data has also been described in detail in our previous article (25). The LC-MS dataset was processed using Progenesis Qi software version 2.3 (Nonlinear Dynamics, Newcastle, UK). Initially, the software conducted sophisticated data mining, incorporating advanced procedures such as alignment, peak selection, normalization, and retention time (RT) correction. The resulting characteristic matrix encapsulates essential details encompassing mass-to-charge ratio (m/z), RT, and peak intensities. Subsequently, metabolite identification was undertaken by leveraging precise m/z values, secondary fragments, and isotope distribution. This process involved querying the Human Metabolome Database (HMDB) (http://www.hmdb.ca/), Lipid Maps (version 2.3) (http://www.lipidmaps.org/), METLIN (http://metlin.scripps.edu/), and internally developed databases (EMDB) for qualitative analysis.

The raw GC-MS data was converted using the software MS-DIAL version 2.74. This software carried out peak detection, peak identification, characterization, peak alignment, wave filtering, etc. Metabolites were characterized using LUG database (Untargeted database of GC–MS rom Lumingbio). The raw data matrix was obtained from the raw data with a three-dimensional dataset, including sample information, the name of the peak of each substance, retention time, retention index, mass-to-charge ratio, and signal intensity, after alignment with the Statistical Compare component. The internal standards with RSD>0.3 were used to segment and normalize all peak signal intensities in each sample, and the segmented and normalized results were removed redundancy and merged peak to obtain the data matrix.

Orthogonal partial least-squares discriminant analysis (OPLS-DA) was used to visualize the differences in metabolites that differed between groups. 200 Response Permutation Testing were used to assess the model’s reliability. The variable importance in projection (VIP) generated in OPLS-DA represented differential metabolites with biological significance. Furthermore, the significance of differential metabolites was further verified by Student’s t test. Variables with VIP > 1.0 and P< 0.05 were considered to be differential metabolites. Metabolic pathway enrichment analysis based on the Kyoto Encyclopedia of Genes and Genomes (KEGG) database (http://www.kegg.jp/kegg/pathway.html).

### 2.4 Machine learning models

Machine learning methodologies were executed utilizing the WEKA Platform (version 3.9.6). A meticulous preprocessing of the dataset was undertaken to generate a balanced sample set, employing the attribute discriminators CfsSubsetEval in conjunction with the BestFirst search method within the software. This approach aimed to discern a subset of metabolites offering optimal predictive capabilities (26). T Subsequently, the assessment of model performance involved the utilization of K-Fold Cross Validation as the testing methodology. Specifically, the dataset was randomized and partitioned into K subsets, one serving as the test set and the remaining as the training set. The learning process entailed extracting features from the training set, while the test machine was employed for prediction. This iterative operation was performed K times to yield K results, and the average of these results was deemed the conclusive outcome. The value of K was deliberately set at 10 to ensure a robust and accurate estimation. In order to find out the best classifiers, we have selected the 24 most commonly used machine learning algorithms (27). Evaluation metrics encompassed the classification true positive rate (TPR), false positive rate (FPR), precision, recall, F-measure, Matthews Correlation Coefficient (MCC), and the area under the receiver operating characteristic curve (AUC).


$$TPR=\frac{TP}{TP+FN}\times 100\%$$

$$FPR=\frac{FP}{FP+TN}\times 100\%$$

$$Precision=\frac{TP}{TP+FP}\times 100\%$$

$$F-Measure=\frac{2\;*\;Precision\;*\;Recall}{Precision+Recall}$$

Where, TPR: true positive rate; TP: true positive; FN: false negative; FPR: false positive rate; FP: false positive; TN: true negative.

## 3 Result

### 3.1 Characteristics of the study population

In our study, a total of 368 individuals were included, comprising 235 subjects who self-reported a diagnosis of hypertension by a physician. Additionally, 49 individuals were diagnosed with depression, out of which 34 were identified as experiencing the coexistence of depression and hypertension. Subjects with hypertension combined with depression served as the HD group, and 34 subjects with hypertension only were matched on the basis of age and sex as the HG group. As shown in **Table 1**, there was no difference between the two groups in terms of age, gender, BMI, and diseases history, except for GDS score.

**Table 1:** Baseline sociodemographic variables of the matched groups (N=68).

Characteristic	HD (N=34)	HC (N=34)	P value
Age (years)	72.41 (± 5.23)	73.59 (± 4.75)	0.355
Sex (%)			0.431
Male	35.3	26.5	
Female	64.7	73.5	
BMI (kg/m2)	24.21 (± 3.18)	24.73 (± 3.74)	0.540
Number of diseases			
Diabetes (%)			0.144
No	70.6	85.3	
Yes	29.4	14.7	
Hyperlipidemia (%)			0.69
No	91.2	88.2	
Yes	8.8	11.8	
Stroke (%)			0.209
No	55.9	70.6	
Yes	44.1	29.4	
Heart disease (%)			0.612
No	61.8	67.6	
Yes	38.2	32.4	
GDS score	14.38 (± 3.04)	4.79 (± 2.53)	<0.001

HD, hypertension with depression groups; HC, hypertension controls; BMI, body mass index, Geriatric Depression Scale score.

### 3.2 Metabolomics results

**Figure 1:**
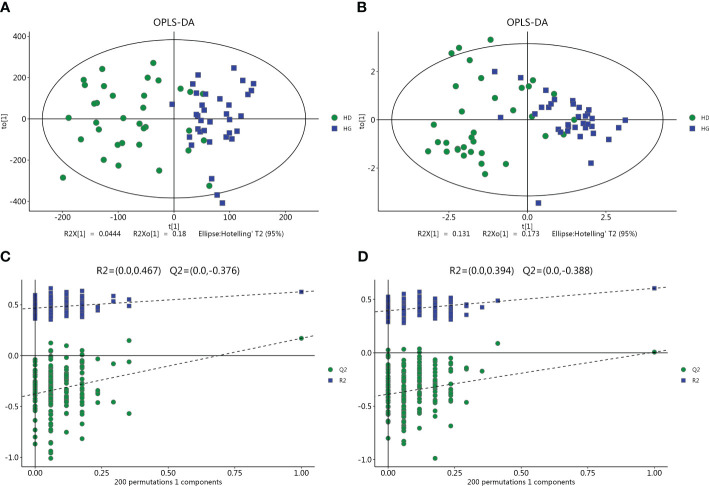
Multivariate date analysis of date from plasma between the hypertension with depression groups (HD) and hypertension controls (HC) base on GC/LC-MS. **(A, C)** OPLS-DA score plots and statistical validation of the corresponding OPLS-DA model by permutation analysis based on the LC-MS. **(B, D)** OPLS-DA score plots and statistical validation of the corresponding OPLS-DA model by permutation analysis based on the GC-MS. The two coordinate points are relatively far away on the score map, indicating that there is a significant difference between the two samples, and vice versa. The elliptical region represents a 95% confidence interval.

**Figure 2:**
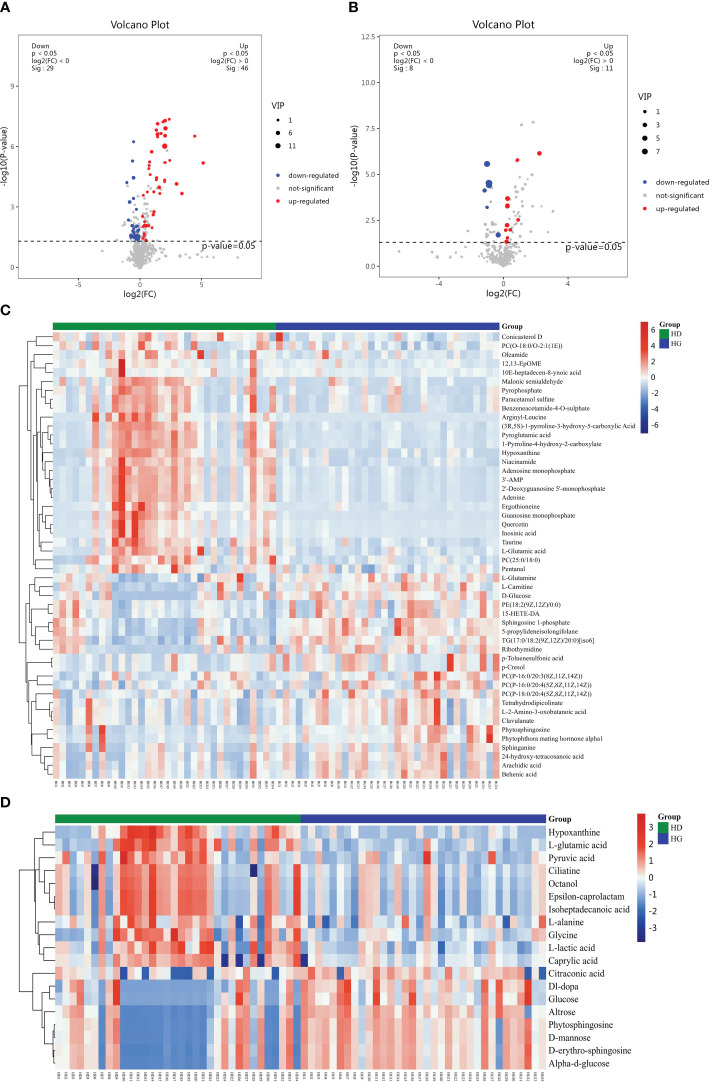
Volcano plot and hierarchical clustering based on the LC/GC-MS of serum metabolites obtained from the depression groups (HD) and hypertension controls (HC). **(A)** Volcano plot based on LC-MS. **(B)** Volcano plot based on GC-MS. **(C)** Hierarchical clustering based on LC-MS. **(D)** Hierarchical Clustering based on GC-MS. In **(A, B)**, the blue dot represents metabolite with a downward trend, red represents metabolites with an upward trend, and the gray origin represents that the change of metabolites is not obvious. The area size of the point is related to the VIP value. In **(C, D)**, the color from blue to red illustrates that metabolites’ expression abundance is low to high in hierarchical clustering.

**Figure 3:**
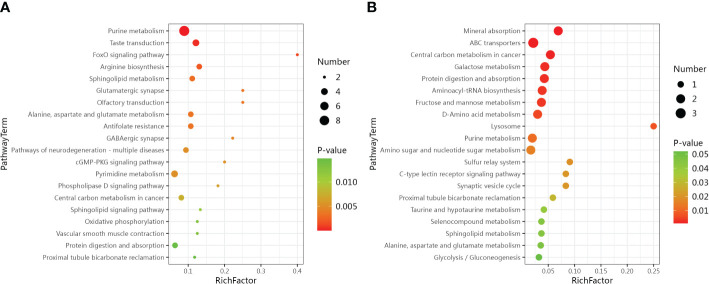
Bubble plots show metabolic pathway enrichment results. **(A)** Metabolic pathway analysis based on LC-MS. **(B)** Metabolic pathway analysis based on GC-MS.

The LC-MS analysis identified 1012 substances, while the GC-MS analysis detected 446 substances. The difference in plasma metabolites between the two groups of samples were evaluated using OPLS‐DA model. The model showed separated and little superimposed between the two groups (**Figures 1A, B**). 200 response permutation tests confirm that the model is reliable (**Figures 1C, D**). Using the VIP value of the first principal component of the OPLS-DA model>1.5 and the p-value of the t-test<0.05 as screening criteria, 65 metabolites detected by LC-MS were considered differential metabolites, while 19 metabolites detected by GC-MS were considered differential metabolites. **Table 2** shows the top 20 metabolites with VIP values. The volcano plots show p-values and fold change values, thus demonstrating the validity of the differential metabolites (**Figures 2A, B**). Hierarchical clustering shows the levels of these metabolites, where the color indicates higher (red) or lower (blue) levels and the intensity reflects the corresponding concentration (**Figures 2C, D**). The metabolic pathway enrichment results indicated that a variety of pathways were altered, with the purine metabolic pathway being the most affected, followed by taste transduction. (**Figure 3**).

**Table 2:** The metabolites with the top 20 VIP values.

Metabolites	VIP^a^	P-value^b^	log2 (FC)	FC^c^	Trend^d^	Method
Hypoxanthine	11.928	<0.001	2.008	4.023	↑	LC-MS
PC(P-18:0/20:4(5Z,8Z,11Z,14Z))	8.964	0.031	-0.159	0.896	↓	LC-MS
D-erythro-sphingosine	7.971	<0.001	-0.880	0.543	↓	GC-MS
L-2-Amino-3-oxobutanoic acid	7.776	0.037	-0.189	0.877	↓	LC-MS
Altrose	7.511	<0.001	-1.016	0.494	↓	GC-MS
Phytosphingosine	7.177	<0.001	-0.897	0.537	↓	GC-MS
D-mannose	7.147	<0.001	-0.921	0.528	↓	GC-MS
Alpha-d-glucose	6.933	<0.001	-0.909	0.532	↓	GC-MS
2’-Deoxyguanosine 5’-monophosphate	6.208	<0.001	2.069	4.197	↑	LC-MS
Citraconic acid	4.819	0.020	-0.325	0.798	↓	GC-MS
p-Toluenesulfonic acid	4.561	0.026	-0.726	0.605	↓	LC-MS
(3R,5S)-1-pyrroline-3-hydroxy-5-carboxylic Acid	4.209	<0.001	1.427	2.689	↑	LC-MS
3’-AMP	3.953	<0.001	2.036	4.100	↑	LC-MS
L-lactic acid	3.818	<0.001	0.253	1.192	↑	GC-MS
Adenosine monophosphate	3.686	<0.001	2.034	4.096	↑	LC-MS
L-Carnitine	3.626	0.027	-0.220	0.858	↓	LC-MS
Malonic semialdehyde	3.505	<0.001	1.560	2.949	↑	LC-MS
Quercetin	3.434	<0.001	2.971	7.842	↑	LC-MS
Sphingosine 1-phosphate	3.431	<0.001	-0.525	0.695	↓	LC-MS
Epsilon-caprolactam	3.382	0.001	0.247	1.186	↑	GC-MS

a Correlation coefficient and VIP value were obtained from OPLS-DA analysis.

b P value determined from Student’s t-test.

c Fold change between hypertension with depression groups and hypertension controls.

d Relative concentrations compared to healthy controls: ↑, upregulated, ↓, downregulated.

FC, fold change; VIP, variable importance for projection.

### 3.3 Machine learning results

The optimal diagnostic value metabolite combination screened with WEKA included 12 metabolites, namely Dl-dopa, glycine, hypoxanthine, 2’-deoxyguanosine 5’-monophosphate, 3’-AMP, malonic semialdehyde, phytosphingosine, conicasterol D, phytophthora mating hormone alpha1, uridine, isopimaric acid and 7-oxo-11E-Tetradecenoic acid. Evaluating the performance of various machine learning algorithms with this combination shows that random forest and bayesnet gave the better results, with better values for various evaluation metrics and ACU values greater than 0.9. The diagnostic performance of Random Forest is superior and it has the best TPR and AUC values (**Table 3**).

**Table 3:** Performance of various algorithms for machine learning.

Classification	Algorithm	TPR	FPR	Precision	Recall	F-Measure	MCC	AUC
Bayes	Bayesnet	0.853	0.147	0.874	0.853	0.851	0.726	0.913
	Naive Bayes	0.794	0.206	0.811	0.794	0.791	0.605	0.860
	NaiveBayesMultinomial	0.750	0.250	0.756	0.750	0.749	0.505	0.804
functions	Logistic	0.691	0.309	0.693	0.691	0.691	0.384	0.770
	MultilayerPerceptron	0.794	0.206	0.798	0.794	0.793	0.592	0.849
	SGD	0.809	0.191	0.822	0.809	0.807	0.631	0.809
	SimpleLogistic	0.750	0.250	0.769	0.750	0.746	0.518	0.798
	SVM	0.809	0.191	0.832	0.809	0.805	0.640	0.809
Lazy	Ibk	0.750	0.250	0.752	0.750	0.750	0.502	0.735
	KStar	0.853	0.147	0.864	0.853	0.852	0.717	0.818
	LWL	0.779	0.221	0.812	0.779	0.773	0.591	0.738
Mate	AdaBoostM1	0.824	0.176	0.834	0.824	0.822	0.657	0.881
	Bagging	0.809	0.191	0.822	0.809	0.807	0.631	0.886
	LogitBoost	0.794	0.206	0.795	0.794	0.794	0.589	0.884
	FilteredClassifier	0.794	0.206	0.794	0.794	0.794	0.588	0.801
	IterativeClassifierOptimizer	0.794	0.206	0.795	0.794	0.794	0.589	0.834
rules	DecisionTable	0.779	0.221	0.782	0.779	0.779	0.561	0.792
	PART	0.750	0.250	0.750	0.750	0.750	0.500	0.762
Tree	DecisionStump	0.779	0.221	0.812	0.779	0.773	0.591	0.715
	HoeffdingTree	0.794	0.206	0.811	0.794	0.791	0.605	0.861
	J48	0.750	0.250	0.752	0.750	0.750	0.502	0.733
	RandomForest	0.853	0.147	0.864	0.853	0.852	0.717	0.932
	RandomTree	0.721	0.279	0.721	0.721	0.721	0.441	0.721
	LMT	0.750	0.250	0.761	0.750	0.747	0.511	0.824

TPR, true positive rate; FPR, false positive rate; MCC, Matthews Correlation Coefficient; AUC, the area under the receiver operating characteristic curve (AUC).

## 4 Discussion

In this study, we used LC-MS and GC-MS to detect metabolites in fasting plasma of subjects and to look for different metabolites of depression in hypertensive subjects. Metabolic pathway enrichment was then used to look for altered metabolic pathways in depressed patients. Using the Weka platform, we carefully selected a subset of metabolites with the best predictive power. We then used this carefully selected subset to identify the most effective machine learning prediction algorithms. We used this combined metabolomics and machine learning approach in order to improve the sensitivity and specificity of the diagnosis of depression in hypertensive patients.

This investigation delineated that random forest and bayesnet emerged as the two cohorts of machine learning algorithms demonstrating superior performance within our study. Both exhibited commendable values, with AUC value surpassing 0.9 for each of the evaluation metrics employed. Presently, machine learning, particularly exemplified by the random forest algorithm, assumed a prominent role in constructing diverse models for predicting disease risks and facilitating disease diagnoses (28). It is worth noting that a previous study only used six machine learning algorithms, among which SVM showed the best predictive performance for depression in hypertensive population (29).This research algorithm is relatively limited and may miss out on algorithms that have good diagnostic effects on individuals with hypertension and depression. Conversely, our findings align with those of Mousavian et al. (30) and de Souza Filho et al. (31) corroborating that Random Forests outperform SVM in depression prediction. Bayesian networks, though, have not consistently exhibited robust performance in antecedent studies, thereby featuring less prominently in the studies (29, 32, 33). The discrepancy may arise from our focus on predictors being differential metabolites in metabolomics, whereas their studies used information from questionnaires, blood markers, or imaging data. Compared to the previous results, we have higher AUC values and better diagnostic performance.

In our study, the metabolites screened by WEKA software with the best predictive value that were most involved in purine metabolism included Hypoxanthine, 2’-Deoxyguanosine 5’-monophosphate and 3’-AMP. Meanwhile, purine metabolism was the most affected pathway. The results of metabolic pathway enrichment showed purine metabolism with Adenosine monophosphate, L-glutamine, inosinic acid, guanosine monophosphate, adenine, hypoxanthine, 2’-deoxyguanosine 5’-monophosphate, xanthine and 3’-AMP. Among these metabolites, only the expression of L-glutamine was up-regulated, while the expression of the remaining metabolites was down-regulated. L-glutamine serves as an important nitrogen donor for de Novo synthesis of both purine and pyrimidine nucleotides (34). In the synthesis of the purine ring, the nitrogen at the 3rd and 9th positions comes from the amide group with glutamine (35). Thus, glutamine can be consumed as a substrate for the synthesis of Adenosine monophosphate, inosinic acid, guanosine monophosphate, adenine, hypoxanthine, 2’-deoxyguanosine 5’-monophosphate, xanthine and 3’-AMP. Glutamine is a precursor of gamma-aminobutyric acid that are important neurotransmitters *in vivo* (36), and affects the transmission of excitation. A previous study found that glutamine was decreased in the prefrontal cortex, hippocampus and amygdala in major depression (37). Ruixin He et al. also found decreased circulating glutamine levels in depressed patients (36).

The subsequent metabolic pathway in our investigation that exhibited a pronounced impact was taste transduction. The metabolites we identified as being involved in taste regulation were AMP, IPM, GMP and L-Glutamate. These metabolites affect the transmission of umami (38, 39). Corroborating our findings, a parallel cross-sectional study conducted in the United States identified an association between depression and discernible alterations in taste (40). Noteworthy in this context is the elucidation of the pathophysiological nexus between taste dysfunction and depression, positing its potential implication in the genesis of anorexia. The latter, being a cardinal symptom of severe depression, manifests in rat models through a discernible diminution in responsiveness to palatable foods. This intricate interplay underscores the multifaceted relationships between mood disorders, sensory perception, and physiological manifestations, enriching our comprehension of the intricate pathways implicated in depressive states (41).

Several studies have indicated alterations in the alanine, aspartate, and glutamate metabolism among individuals with hypertension (42). Intriguingly, our investigation demonstrated metabolic enrichment highlighting alterations in the alanine, aspartate, and glutamate metabolism among patients solely diagnosed with hypertension, contrasting with those presenting both hypertension and depression. The alanine, aspartate, and glutamate metabolism serves as a link between hypertension and depression. Hence, this pathway presents significant potential for elucidating the causal relationship between hypertension and depression, as well as for devising treatment strategies for patients suffering from both conditions. This suggests a potential avenue for treating refractory depression.

## 5 Limitations

While our investigation has yielded valuable insights into advancing the diagnosis of depression and elucidating underlying mechanisms, it is imperative to acknowledge certain limitations. It is well documented that metabolomics studies have a sample size of no less than 20 per group (43). We had 34 individuals in each group, for a total sample size of 68, which meets the needs of metabolomics studies. However, with a larger sample size, more interesting features may be found. Our study population was drawn only from older adults aged 65 years and older with a mono-dietary pattern in Chongming, Shanghai, which limits the applicability of our findings to a wider population. In order to enhance the reliability and generalisability of our study and to reduce bias due to small sample size, we are recruiting more subjects in multiple locations to participate in our study. A secondary constraint pertains to the absence of direct validation of our results, notwithstanding corroboration gleaned from extant literature. This methodological refinement aligns with our commitment to methodological rigor and the fortification of the scientific foundation underpinning our investigative endeavors. In future studies, we increase the validation group, and we also conduct animal experiments or cellular experiments to discover the pathogenesis of depression.

## 6 Conclusion

This study demonstrates that metabolites and metabolic pathways are altered in older adults with hypertension combined with depression compared to older adults with hypertension alone. Methods using metabolomics and machine learning excelled in predicting depression in hypertensive older adults. This approach helps in diagnosing depression in hypertensive patients.

## Data availability statement

The datasets generated during and analyzed during the current study are not publicly available due to protect study participant privacy but are available from the corresponding author on reasonable request. Requests to access these datasets should be directed to QG, guoq@sumhs.edu.cn.

## Ethics statement

The studies involving humans were approved by Shanghai University of Medicine and Health Sciences, China. The studies were conducted in accordance with the local legislation and institutional requirements. The participants provided their written informed consent to participate in this study.

## Author contributions

JG: Conceptualization, Writing – original draft, Writing – review & editing. JZ: Conceptualization, Writing – original draft, Writing – review & editing. PH: Writing – review & editing, Data curation, Funding acquisition. YhW: Data curation, Writing – review & editing. KZ: Data curation, Writing – review & editing. CH: Data curation, Writing – review & editing. YW: Data curation, Writing – review & editing. CC: Data curation, Writing – review & editing. QG: Writing – review & editing, Funding acquisition.
