# Supplementary material for: Finding the best predictive model for hypertensive depression in older adults based on machine learning and metabolomics research
Source: Front Psychiatry. 2024 Jun 27;15:1370602. doi: 10.3389/fpsyt.2024.1370602 (PMC11236531; doi:10.3389/fpsyt.2024.1370602)
Supplement: Supplementary file 1 [file DataSheet_1.pdf]

S1.All differential metabolites detected.

Metabolites	KEGG	VIP	P-value	log2(FC)	FC	Trend	Methor
D-erythro-sphingosine		7.971	<0.001	-0.880	0.543	↓	GC-MS
Altrose		7.511	<0.001	-1.016	0.494	↓	GC-MS
Phytosphingosine	C12144	7.177	<0.001	-0.897	0.537	↓	GC-MS
D-mannose	C00159	7.147	<0.001	-0.921	0.528	↓	GC-MS
Alpha-d-glucose	C00267	6.933	<0.001	-0.909	0.532	↓	GC-MS
Citraconic acid		4.819	0.020	-0.325	0.798	↓	GC-MS
L-lactic acid		3.818	<0.001	0.253	1.192	↑	GC-MS
Epsilon-caprolactam	C06593	3.382	0.001	0.247	1.186	↑	GC-MS
Isoheptadecanoic acid		3.367	<0.001	0.246	1.186	↑	GC-MS
Octanol		3.326	0.006	0.225	1.168	↑	GC-MS
Hypoxanthine	C00262	3.072	<0.001	2.226	4.678	↑	GC-MS
Ciliatine		2.679	0.046	0.189	1.140	↑	GC-MS
Dl-dopa		2.532	<0.001	-1.169	0.445	↓	GC-MS

Pyruvic acid		1.622	0.003	0.907	1.875	↑	GC-MS
Glycine	C00037	1.304	0.010	0.433	1.350	↑	GC-MS
L-glutamic acid		1.302	<0.001	0.871	1.829	↑	GC-MS
L-alanine	C00041	1.261	0.011	0.141	1.103	↑	GC-MS
Caprylic acid		1.060	0.028	0.247	1.187	↑	GC-MS
Glucose		1.056	<0.001	-1.016	0.495	↓	GC-MS
Hypoxanthine	C00262	11.928	<0.001	2.008	4.023	↑	LC-MS
PC(P-18:0/20:4(5Z,8Z,11Z,14Z))		8.964	0.031	-0.159	0.896	↓	LC-MS
L-2-Amino-3-oxobutanoic acid	C03508	7.776	0.037	-0.189	0.877	↓	LC-MS
2'-Deoxyguanosine 5'-monophosphate	C00362	6.208	<0.001	2.069	4.197	↑	LC-MS
p-Toluenesulfonic acid	C06677	4.561	0.026	-0.726	0.605	↓	LC-MS
(3R,5S)-1-pyrroline-3-hydr	C04281	4.209	<0.001	1.427	2.689	↑	LC-MS

oxy-5-carboxylic Acid							
3'-AMP	C01367	3.953	<0.001	2.036	4.100	↑	LC-MS
Adenosine monophosphate	C00020	3.686	<0.001	2.034	4.096	↑	LC-MS
L-Carnitine	C00318	3.626	0.027	-0.220	0.858	↓	LC-MS
Malonic semialdehyde	C00222	3.505	<0.001	1.560	2.949	↑	LC-MS
Quercetin	C00389	3.434	<0.001	2.971	7.842	↑	LC-MS
Sphingosine 1-phosphate	C06124	3.431	<0.001	-0.525	0.695	↓	LC-MS
D-Glucose	C00221	3.254	<0.001	-0.841	0.558	↓	LC-MS
12,13-EpOME	C14826	3.193	0.009	0.532	1.446	↑	LC-MS
PE(18:2(9Z,12Z)/0:0)		3.110	0.044	-0.258	0.836	↓	LC-MS
PC(P-16:0/20:3(8Z,11Z,14Z))		3.084	0.047	-0.216	0.861	↓	LC-MS
Taurine	C00245	3.084	<0.001	0.950	1.932	↑	LC-MS
Pyroglutamic	C01879	2.999	<0.001	1.436	2.705	↑	LC-MS

acid							
Phytosphingosine	C12144	2.974	0.009	-0.630	0.646	↓	LC-MS
15-HETE-DA		2.840	0.037	-0.294	0.816	↓	LC-MS
Inosinic acid	C00130	2.839	<0.001	3.404	10.586	↑	LC-MS
Clavulanate	C06662	2.708	0.033	-0.197	0.872	↓	LC-MS
Paracetamol sulfate		2.649	0.002	1.139	2.202	↑	LC-MS
Arginyl-Leucine		2.542	<0.001	5.132	35.075	↑	LC-MS
1-Pyrroline-4-hydroxy-2-carboxylate	C04282	2.342	<0.001	1.329	2.512	↑	LC-MS
10E-heptadecen-8-ynoic acid		2.198	0.045	0.492	1.407	↑	LC-MS
Arachidic acid	C06425	2.196	0.026	-0.425	0.745	↓	LC-MS
Behenic acid	C08281	2.196	0.019	-0.488	0.713	↓	LC-MS
Benzeneacetamide-4-O-sul		2.072	0.002	1.075	2.106	↑	LC-MS

phate							
Sphinganine	C00836	2.031	0.035	-0.384	0.767	↓	LC-MS
TG(17:0/18:2(9Z,12Z)/20:0)[iso6]		1.858	<0.001	-0.527	0.694	↓	LC-MS
PC(25:0/18:0)		1.856	<0.001	1.353	2.554	↑	LC-MS
Conicasterol D		1.845	0.008	0.364	1.287	↑	LC-MS
Tetrahydrodipicolinate	C03972	1.825	0.015	-0.151	0.901	↓	LC-MS
PC(P-16:0/20:4(5Z,8Z,11Z,14Z))		1.768	0.009	-0.225	0.856	↓	LC-MS
5-propylidene isolongifolane		1.685	<0.001	-0.604	0.658	↓	LC-MS
Pyrophosphate	C00013	1.653	<0.001	1.125	2.181	↑	LC-MS
Niacinamide	C00153	1.574	<0.001	1.721	3.296	↑	LC-MS
L-Glutamine	C00064	1.570	0.017	-0.184	0.880	↓	LC-MS
Phytophthora mating hormone		1.513	0.004	-0.934	0.523	↓	LC-MS

alpha1							
L-Glutamic acid	C00025	1.472	<0.001	0.782	1.720	↑	LC-MS
Guanosine monophosphate	C00144	1.471	<0.001	4.448	21.831	↑	LC-MS
Pentanal		1.467	0.003	0.209	1.156	↑	LC-MS
Ribothymidine		1.460	<0.001	-1.068	0.477	↓	LC-MS
Ergothioneine	C05570	1.422	<0.001	2.003	4.009	↑	LC-MS
Oleamide	C19670	1.414	0.009	0.719	1.646	↑	LC-MS
p-Cresol	C01468	1.397	0.032	-0.744	0.597	↓	LC-MS
Adenine	C00147	1.376	<0.001	2.014	4.040	↑	LC-MS
24-hydroxy-tetracosanoic acid		1.363	0.016	-0.583	0.668	↓	LC-MS
PC(O-18:0/O-2:1(1E))		1.335	0.021	0.335	1.262	↑	LC-MS
20a,22b-Dihydroxycholesterol	C05501	1.317	0.035	0.253	1.192	↑	LC-MS
24-methylene		1.295	0.010	-0.335	0.793	↓	LC-MS

-cholest-5-en-3beta,7beta,19-triol							
omega-hydroxy behenic		1.258	0.032	-0.493	0.710	↓	LC-MS
15(S)-HETE	C04742	1.236	<0.001	1.612	3.057	↑	LC-MS
Uridine	C00299	1.226	<0.001	-0.517	0.699	↓	LC-MS
Xeniasterol-b		1.224	0.026	0.350	1.275	↑	LC-MS
Dihydro-2(3H)-thiophenone		1.164	0.001	-0.293	0.816	↓	LC-MS
(-)-alpha-Bisabolol	C09621	1.128	<0.001	0.597	1.513	↑	LC-MS
Isopimaric acid	C09118	1.119	<0.001	1.431	2.697	↑	LC-MS
Xanthine	C00385	1.104	<0.001	1.864	3.641	↑	LC-MS
27-Norcholestanehexol		1.102	0.009	0.463	1.379	↑	LC-MS
Hydroxypropionic acid	C01013	1.097	<0.001	0.272	1.208	↑	LC-MS
18-fluoro-9Z,12Z-octadecadienoic acid		1.090	<0.001	1.327	2.509	↑	LC-MS

Dimethylglycine	C01026	1.085	<0.001	0.736	1.666	↑	LC-MS
1-(sn-Glycero-3-phospho)-1D-myo-inositol	C01225	1.074	<0.001	2.378	5.200	↑	LC-MS
Erucic acid	C08316	1.072	0.012	-0.513	0.701	↓	LC-MS
Undecanal		1.067	<0.001	0.828	1.776	↑	LC-MS
Glycylproline		1.055	0.014	-0.087	0.941	↓	LC-MS
CDP-Ethanolamine	C00570	1.047	<0.001	2.416	5.338	↑	LC-MS
PC(16:1(9Z)/2:0)		1.040	0.011	0.980	1.973	↑	LC-MS
3-Oxoglutaric acid		1.035	0.005	0.372	1.294	↑	LC-MS
14,15-Epoxy-5,8,11-eicosatrienoic acid	C14771	1.032	<0.001	1.998	3.995	↑	LC-MS
7-oxo-11E-Tetradecenoic acid		1.018	0.009	0.270	1.206	↑	LC-MS
Fumaric acid	C00122	1.011	<0.001	0.717	1.644	↑	LC-MS

8,9-Epoxyeicosatrienoic acid	C14769	1.011	<0.001	2.047	4.132	↑	LC-MS
